# A novel immune-related gene signature predicting survival in sarcoma patients

**DOI:** 10.1016/j.omto.2021.12.007

**Published:** 2021-12-09

**Authors:** Haoyu Ren, Alexandr V. Bazhin, Elise Pretzsch, Sven Jacob, Haochen Yu, Jiang Zhu, Markus Albertsmeier, Lars H. Lindner, Thomas Knösel, Jens Werner, Martin K. Angele, Florian Bösch

**Affiliations:** 1 Department of General, Visceral, and Transplant Surgery, Ludwig-Maximilians-University Munich, 81377 Munich, Germany; 2 German Cancer Consortium (DKTK), Partner Site Munich, Munich, Germany; 3 Department of Liver Surgery and Liver Transplantation Centre, State Key Laboratory of Biotherapy and Cancer Center, West China Hospital, Sichuan University, Chengdu, China; 4 Department of Medicine III, SarKUM, University Hospital, LMU Munich, Munich, Germany; 5 Institute of Pathology, University Hospital, LMU Munich, Munich, Germany

**Keywords:** sarcoma, gene signature, immune infiltration, immunotherapy, prognosis

## Abstract

Sarcomas are a heterogeneous group of rare mesenchymal tumors. The migration of immune cells into these tumors and the prognostic impact of tumor-specific factors determining their interaction with these tumors remain poorly understood. The current risk stratification system is insufficient to provide a precise survival prediction and treatment response. Thus, valid prognostic models are needed to guide treatment. This study analyzed the gene expression and outcome of 980 sarcoma patients from seven public datasets. The abundance of immune cells and the response to immunotherapy was calculated. Immune-related genes (IRGs) were screened through a weighted gene co-expression network analysis (WGCNA). A least absolute shrinkage and selection operator (LASSO) Cox regression was used to establish a powerful IRG signature predicting prognosis. The identified IRG signature incorporated 14 genes and identified high-risk patients in sarcoma cohorts. The 14-IRG signature was identified as an independent risk factor for overall and disease-free survival. Moreover, the IRG signature acted as a potential indicator for immunotherapy. The nomogram based on the risk score was built to provide a more accurate survival prediction. The decision tree with IRG risk score discriminated risk subgroups powerfully. This proposed IRG signature is a robust biomarker to predict outcomes and treatment responses in sarcoma patients.

## Graphical abstract

**Figure:**
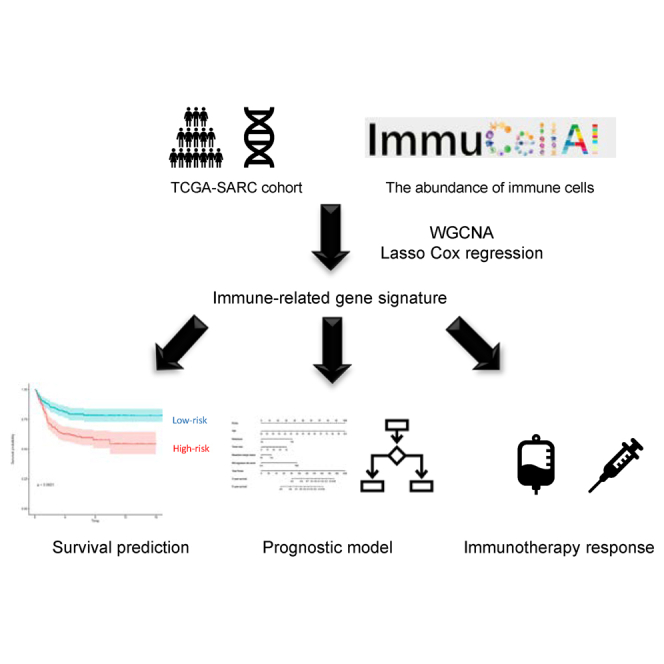

## Teaser

Immune-related gene (IRG) signature serves as a robust and independent prognostic biomarker in various sarcoma subtypes and guides more effective immunotherapy strategies. The nomogram and the decision tree based on the IRG signature could be useful predictive tools and could help to identify high-risk sarcoma patients.

## Introduction

Sarcomas arise from the skeleton and the soft tissue subdividing in various histologic subtypes and have an increasing incidence of 7.7 cases/100,000 individuals per year.^1^^,^^2^ In the European Union, about 27,908 new cases per year are registered.^3^^,^^4^ The therapeutic approaches differ between the subgroups, but surgery offers the only chance of cure.^5^^,^^6^ However, in large series, recurrence rates are as high as up to 45%, which underlines the importance of a precise diagnostic and therapeutic workup.^7^^,^^8^ In this respect, the anatomic heterogeneity complicates the standardization of diagnosis and therapy. Currently the main prognostic criteria for sarcomas are tumor grade, size, histological subtype, and resection margin status,^9^ which makes it challenging to precisely assess the individual prognosis. Consequently, it is imperative to develop robust biomarkers to predict the prognosis and the therapeutic response of sarcoma patients.

Increasing evidence suggests that tumor-infiltrating immune cells determine the clinical outcome of immunotherapy in different sarcoma subtypes.^10^, ^11^, ^12^, ^13^ Immunosurveillance is mediated in part by two major groups of T cells (CD4+, CD8+), which play a pivotal role in tumor formation, tumor progression, and therapy.^14^ Moreover, previous studies demonstrated that an abundance of specific T cell subsets exerts favorable effects on the immune status of patients, improving responses to anticancer treatment and, subsequently, prognosis.^15^^,^^16^ However, although it is still not clear which genes regulate the microenvironment of sarcomas and the abundance of tumor-infiltrating lymphocytes (TILs), it is reasonable to assume that immune-cell-related genes influence the prognosis of sarcoma patients. In this regard, recently an accurate and unique method called Immune Cell Abundance Identifier (ImmuCellAI) has been implemented. ImmuCellAI allows us to calculate the abundance of 24 tumor-related immune cell types from gene expression data,^17^ which enables analyzing the association between the abundance of immune cells, the underlying genes, and the prognosis of cancer patients. Moreover, ImmuCellAI seems to facilitate an accurate prediction of the response to immunotherapy in cancer patients.^17^

Thus, the present study aimed to develop a prognostically relevant immune-cell-related gene signature based on sarcoma-related data from The Cancer Genome Atlas (TCGA-SARC) and validate this signature in six independent sarcoma cohorts. In addition, we aimed to analyze the association between immunological features and the gene signature to predict the response to immunotherapy in the sarcoma cohorts. Finally, based on the gene signature and clinicopathological data, we sought to establish a novel nomogram and decision tree to improve risk stratification in daily clinical practice.

## Results

### Flow chart of the study design

**Figure 1:**
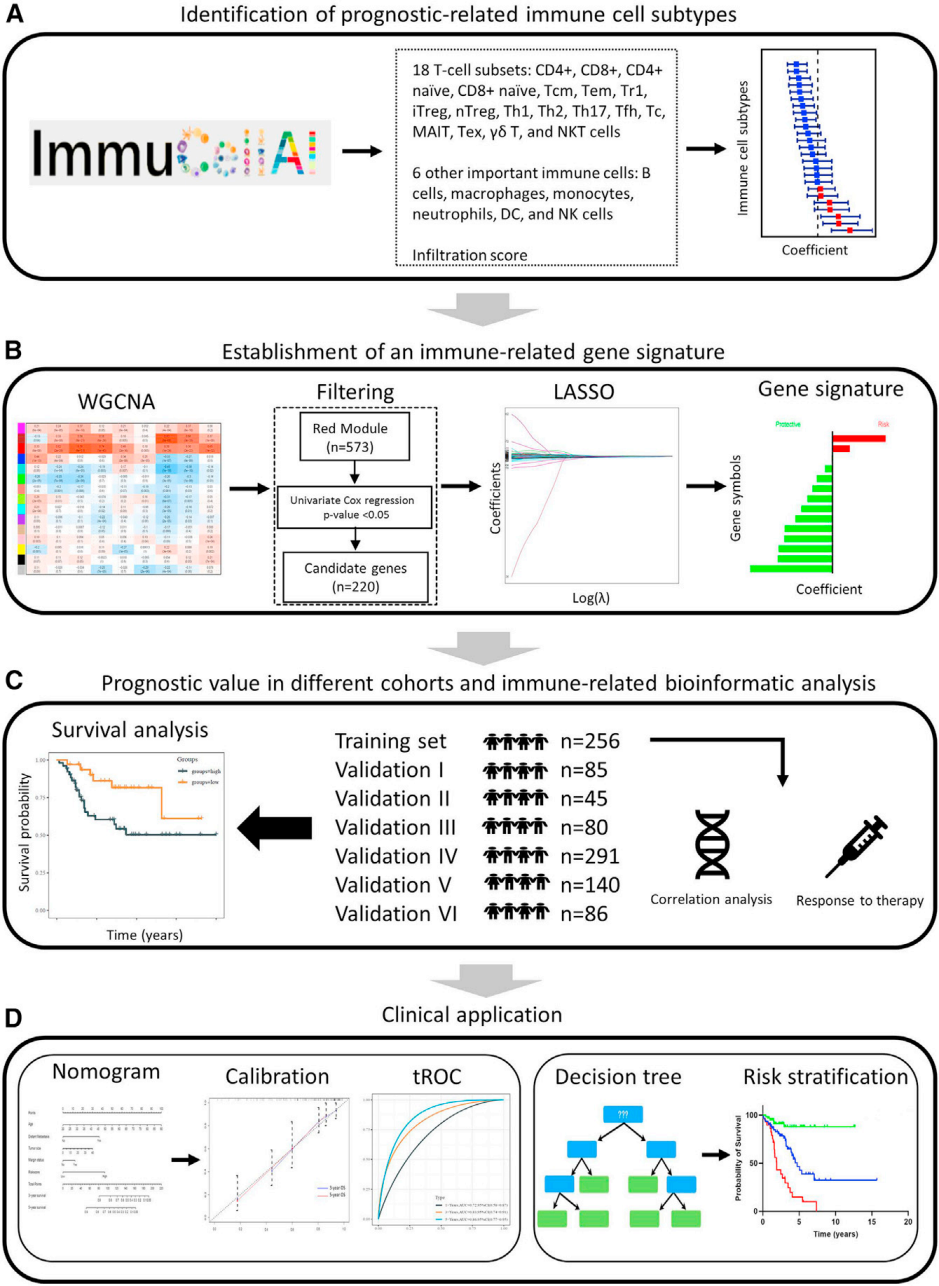
Flow chart of the study design (A) Identification of the immune cell subtypes with prognostic relevance. (B) The approaches used to establish an immune-related gene signature for prognosis. (C) The prognostic value of the gene signature was investigated in different cohorts. (D) This application of personalized medicine might be used in daily routine. WGCNA, weighted gene co-expression network analysis; tROC, time-dependent receiver operating characteristic.

First, the abundance scores of 24 immune cell types and the infiltration scores were downloaded from the ImmuCellAI database and transformed into a *Z* score using the scale method in R project. The univariate Cox proportional regression analysis was used to identify the protective immune cell types that promote a better prognosis in TCGA-SARC patients (Figure 1A). Then, a weighted gene co-expression network analysis (WGCNA) was performed to select the gene module most relevant to these protective immune cell subtypes. In total, 573 genes were extracted from the “MEred module” and further analyzed by univariate Cox regression and LASSO Cox regression analyses. As a result, 14 immune-related prognostic genes were identified and developed into a multiple gene signature with the corresponding coefficients (Figure 1B). Subsequently, the prognostic value of this immune-related gene (IRG) signature was investigated in the TCGA-SARC cohort and six independent validation cohorts. Moreover, the immunologic features and the response to immunotherapy were also explored in high- and low-risk groups based on the signature risk score (Figure 1C). In order to apply the gene signature to clinical practice, the nomogram and decision tree combined clinical information, and IRG risk scores were established to determine the survival outcome and risk levels for sarcoma patients (Figure 1D).

### Prognostic relevance of T cell subtypes and NK cells

**Figure 2:**
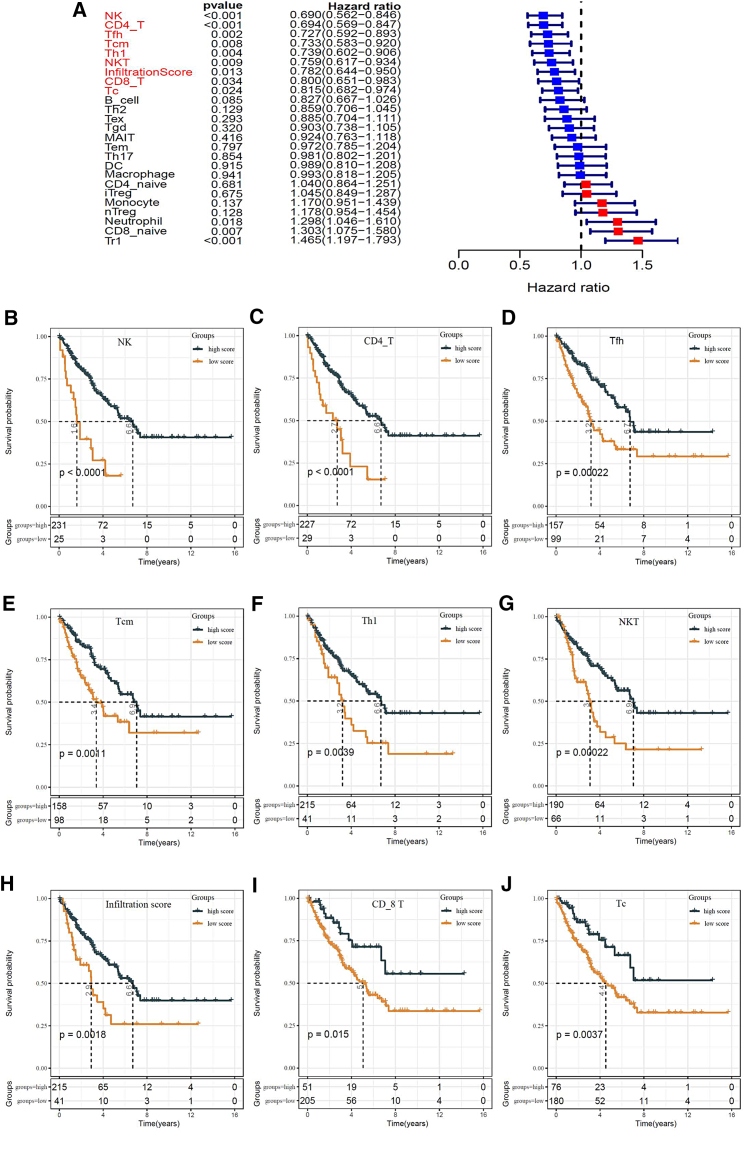
Immune cells were identified as the primary protective factors for survival (A) Univariate Cox regression analysis showed that 7 T cell types, NK cells, and the infiltration score were protective factors among the scores of various immune cell subsets. (B–H) Kaplan–Meier analysis indicated that patients with higher scores of NK (B), CD4+ T (C), Tfh (D), Tcm (E), Th1 (F), NKT (G), CD8+ T (I), and Tc (J) cells and infiltration score (H) had better OS (p < 0.05). NK, natural killer cells; Tfh, follicular helper T cells; Tcm, central memory T cells; Th1, T helper type 1 cells; NKT, natural killer T cells; Tc, cytotoxic T cells.

The univariate Cox regression analysis demonstrated that the abundance scores of eleven immune cells and the infiltration score were significantly associated with overall survival (OS) in the training set. Nine of these scores, including natural killer (NK), CD4+ T, T follicular helper (Tfh), central memory T (Tcm), T helper type 1 (Th1), natural killer T (NKT), CD8+ T, cytotoxic T cell (Tc), and the infiltration score, correlated positively with survival rates (Figure 2A). The 256 patients of the TCGA-SARC cohort were divided into high-score and low-score groups based on the optimal immune-score-cut-off value, and the high-score group exhibited a better prognosis than the low-score group (Figures 2B–2J).

### Construction of the IRG signature for OS

**Figure 3:**
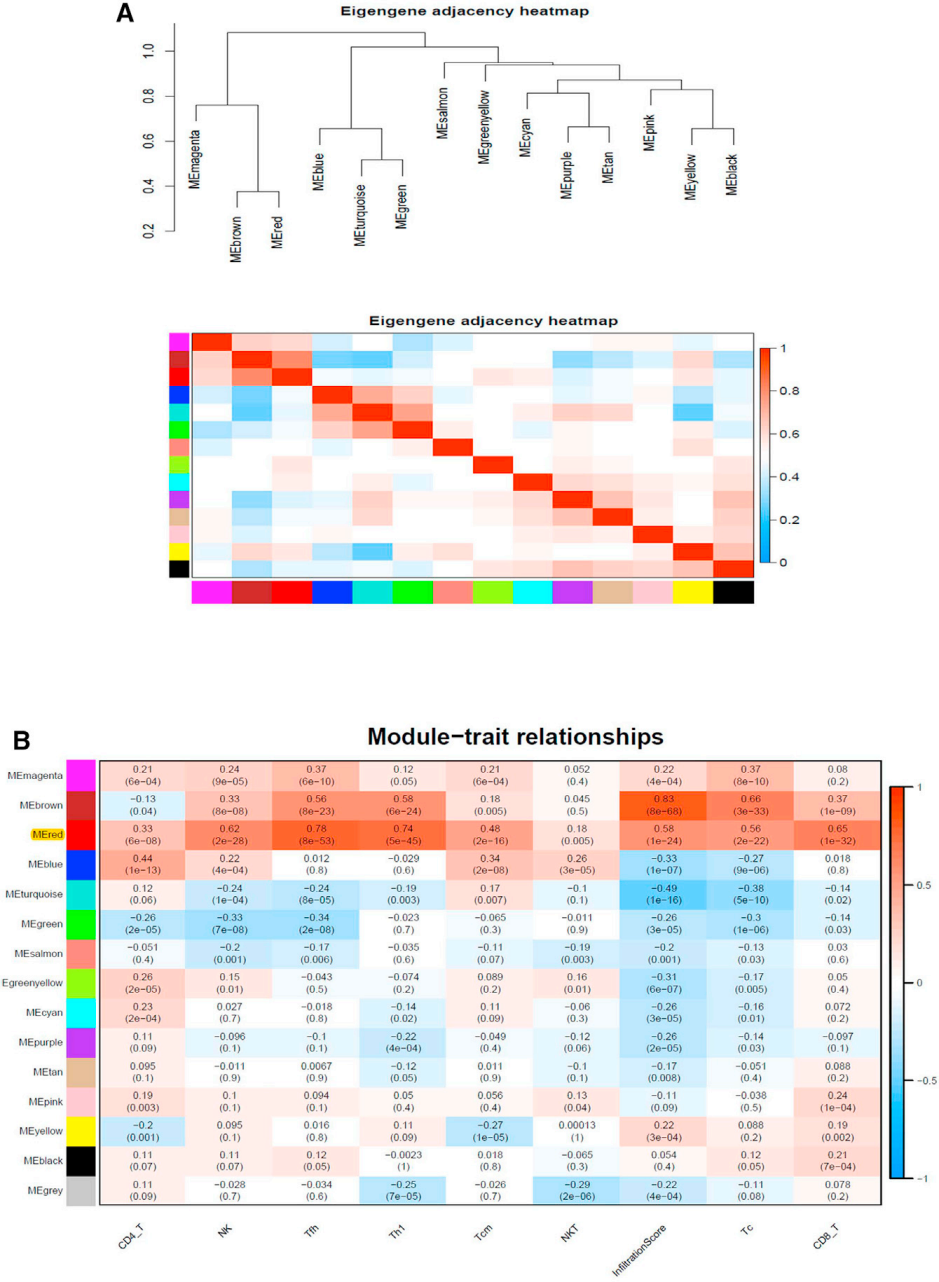
The construction of co-expression modules and module-trait relationships of sarcomas (A) Visualizing the gene network using a heatmap plot. (B) Correlation of module eigengenes with all the protective immune cell scores. Each unit contains the corresponding correlation coefficient and p value.

**Figure 4:**
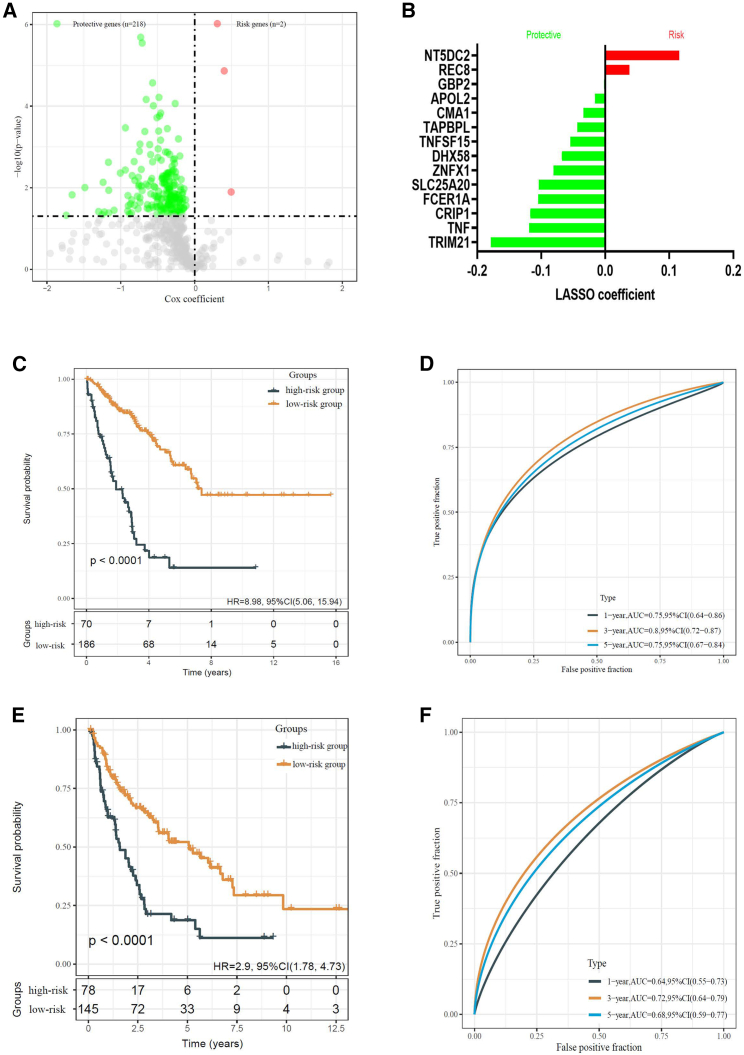
Establishment of the immune-related gene signature (A) A total of 220 prognosis-related candidates were identified among 573 genes extracted from the red module (“MEred”). (B) Gene symbols and the corresponding LASSO coefficients of the IRG signature. (C) Kaplan–Meier curve revealed that patients with higher IRG risk scores exhibited worse OS. (D) The time-dependent ROC curves of the prognostic signature for OS in the training cohort. IRG, immune-related gene; LASSO, least absolute shrinkage and selection operator; ROC, receiver operating characteristic. (E) Kaplan–Meier analysis indicated that patients with higher IRG risk scores showed shorter DFS. (F) The time-dependent ROC curves of the prognostic signature for DFS in the training cohort.

A WGCNA was performed to identify the most significant gene modules related to the nine protective immune scores. When β = 5 (a soft threshold), the scale-free R2 was 0.85, generating a scale-free co-expression network (Figures S1A and S1B), and a total of 14 non-gray modules were obtained (black, blue, brown, cyan, green, green-yellow, magenta, pink, purple, red, salmon, tan, turquoise, and yellow) (Figures S1C and 3A). Among these 14 gene co-expression modules, the red module (“MEred module”) had the most significant correlation with all nine protective immune scores (Figure 3B). Subsequently, all genes from the red module were further analyzed by a univariate Cox regression, and thereby 220 prognosis-related genes were identified. Most of these genes exerted a protective effect, and only two genes were negatively correlated (Figure 4A).

A LASSO Cox regression analysis was applied to identify the most robust prognostic genes and the corresponding coefficients (Figures S2A, S2B, and 4B). This analysis revealed 14 prognostically relevant genes (NT5DC2, REC8, GBP2, APOL2, CMA1, TAPBPL, TNFSF15, DHX58, ZNFX1, SLC25A20, FCER1A, CRIP1, TNF, TRIM21). Thus, the risk score formula of the IRG signature reads as follows: NT5DC2 × 0.11572 + REC8 × 0.037668 + GBP2 × (−0.00153) + APOL2 × (−0.01601) + CMA1 × (−0.0341) + TAPBPL × (−0.04386) + TNFSF15 × (−0.05468) + DHX58 × (−0.06778) + ZNFX1 × (−0.08096) + SLC25A20 × (−0.10396) + FCER1A × (−0.10488) + CRIP1 × (−0.11699) + TNF × (−0.11901) + TRIM21 × (−0.17847). The patients of the training set were then divided into a high- and low-risk groups according to the optimal cut-off value generated from the IRG risk score formula. The high-risk group had a highly significant decreased OS compared with the low-risk group (p < 0.0001, hazard ratio [HR] = 8.98, 95% CI: 5.06–15.94; Figure 4C). Moreover, the areas under the curve (AUCs) of the IRG signature for 1-, 3-, and 5-year OS were 0.75, 0.8, and 0.75, respectively (Figure 4D). In addition, the disease-free survival (DFS) of high-risk patients was also highly significantly shorter than that of low-risk patients (p < 0.0001, HR = 2.9, 95% CI: 1.78–4.73; Figure 4E). The AUCs of 1-, 3-, and 5-year DFS were 0.64, 0.72, and 0.68, respectively (Figure 4F).

**Figure 5:**
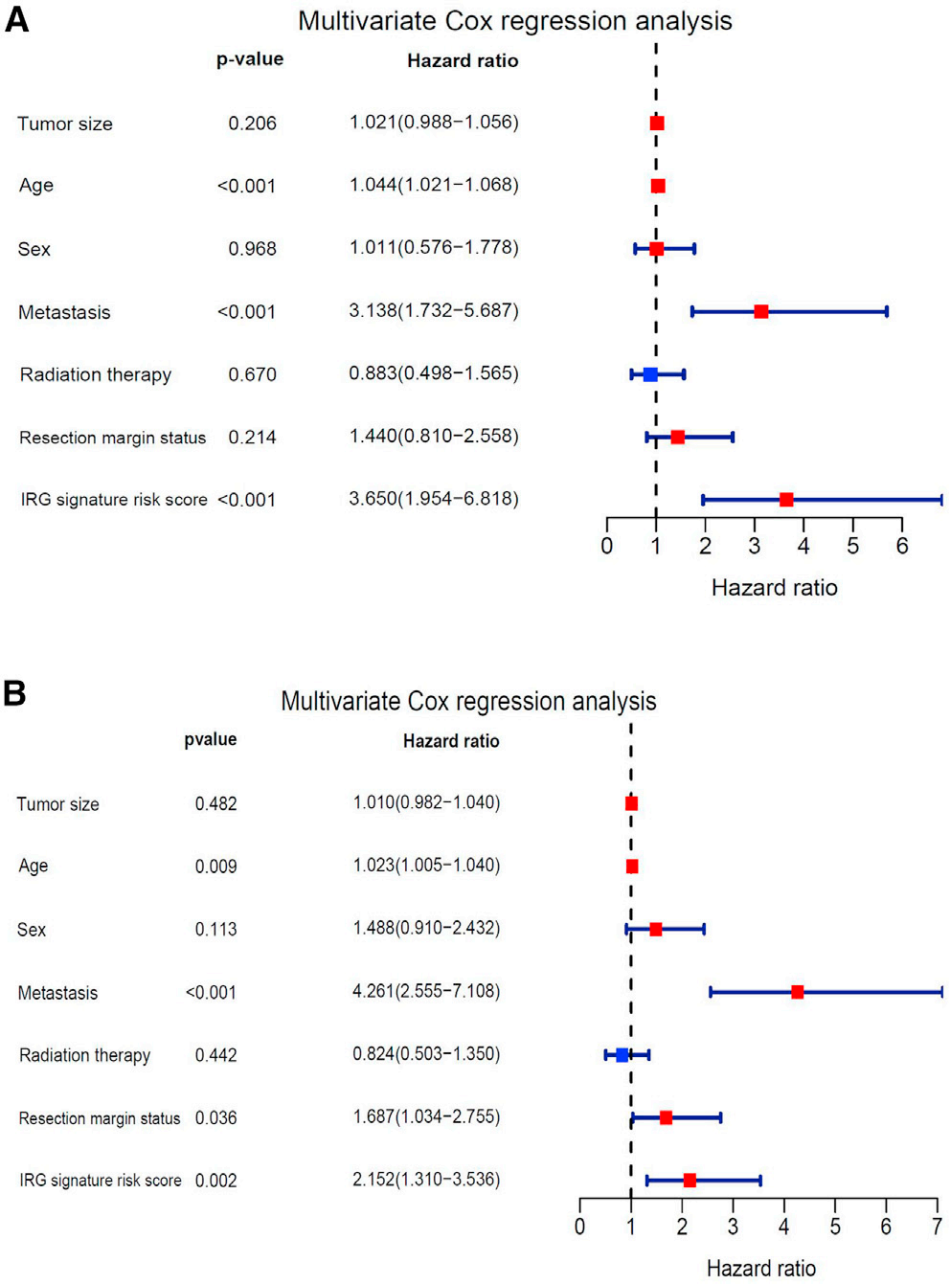
Independent prognostic factors for OS and DFS in the TCGA-SARC cohort (A and B) Multivariate Cox regression analysis of the relationship between clinicopathological features (including the risk score) and OS (A)/DFS (B) of patients in the TCGA-SARC dataset.

To confirm whether the IRG signature was an independent prognostic factor, multivariate Cox regression analyses were performed in the patients (150 patients for OS and 133 patients for DFS) of the TCGA-SARC cohort with complete clinicopathological information. The multivariate Cox regression analysis showed that age, metastasis, and IRG signature risk score were significantly associated with OS (Figure 5A). Additionally, age, metastasis, resection margin status, and IRG signature risk score were proved to be independent risk factors for DFS (Figure 5B).

### Validation of the IRG signature in six independent sarcoma cohorts

To determine the robustness of the identified 14 IRG signatures, validation survival analyses were performed in six independent sarcoma cohorts. The patients of these six validation groups were divided into high- and low-risk groups according to the generated risk score formula, and thereby the Kaplan–Meier survival analyses proved the prognostic relevance of the IRG signature in all six independent validation groups.

**Figure 6:**
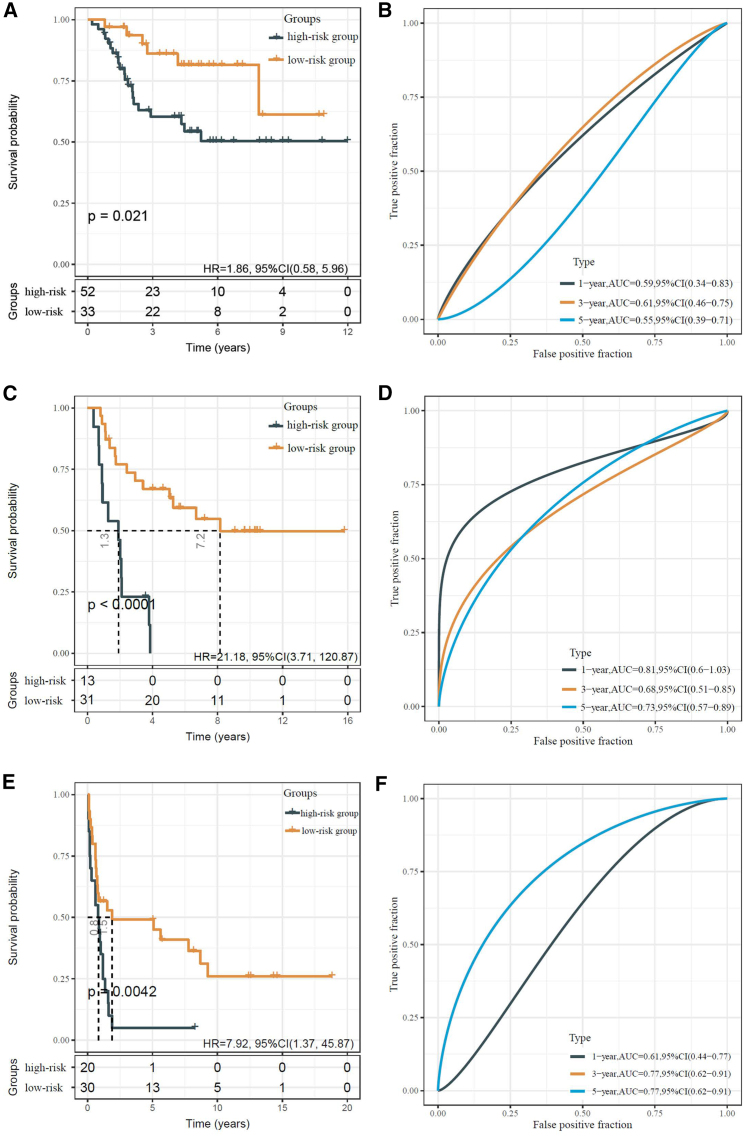
Validation of the OS-related prognostic relevance of the IRG signature with three external datasets (A) Kaplan–Meier analysis of OS in the TARGET-Osteosarcoma cohort (validation cohort I). (B) The AUC of the risk score predicting the 1-, 3-, and 5-year OS in the TARGET-Osteosarcoma cohort. (C) Kaplan–Meier analysis of OS in the GEO: GSE17674 cohort (validation cohort II). (D) The AUC of the risk score predicting the 1-, 3-, and 5-year OS in the GEO: GSE17674 cohort. (E) Kaplan–Meier analysis of OS in the GEO: GSE119041 cohort (validation cohort III). (F) The AUC of the risk score predicting the 1-, 3-, and 5-year OS in the GEO: GSE119041 cohort.

The OS of the high-risk groups was significantly worse compared with the low-risk groups of the validation cohorts I–III (TARGET-Osteosarcoma: HR = 1.86, 95% CI: 0.58–5.96, p = 0.021; GEO: GSE17674: HR = 21.18, 95% CI: 3.71–120.87, p < 0.0001; GEO: GSE119041: HR = 7.92, 95% CI: 1.37–45.87, p = 0.0042; Figures 6A, 6C, and 6E, respectively). The receiver-operating characteristic (ROC) curve analysis of the IRG signature showed a beneficial 1-, 3-, and 5-year AUC in each cohort (TARGET-Osteosarcoma: AUCs of 1-, 3-, and 5-year OS were 0.59, 0.61, and 0.55, respectively; GEO: GSE17674: AUCs of 1-, 3-, and 5-year OS were 0.81, 0.68, and 0.73, respectively; GEO: GSE119041: AUCs of 1-, 3-, and 5-year OS were 0.61, 0.77, and 0.77, respectively; Figures 6B, 6D, and 6F, respectively).

**Figure 7:**
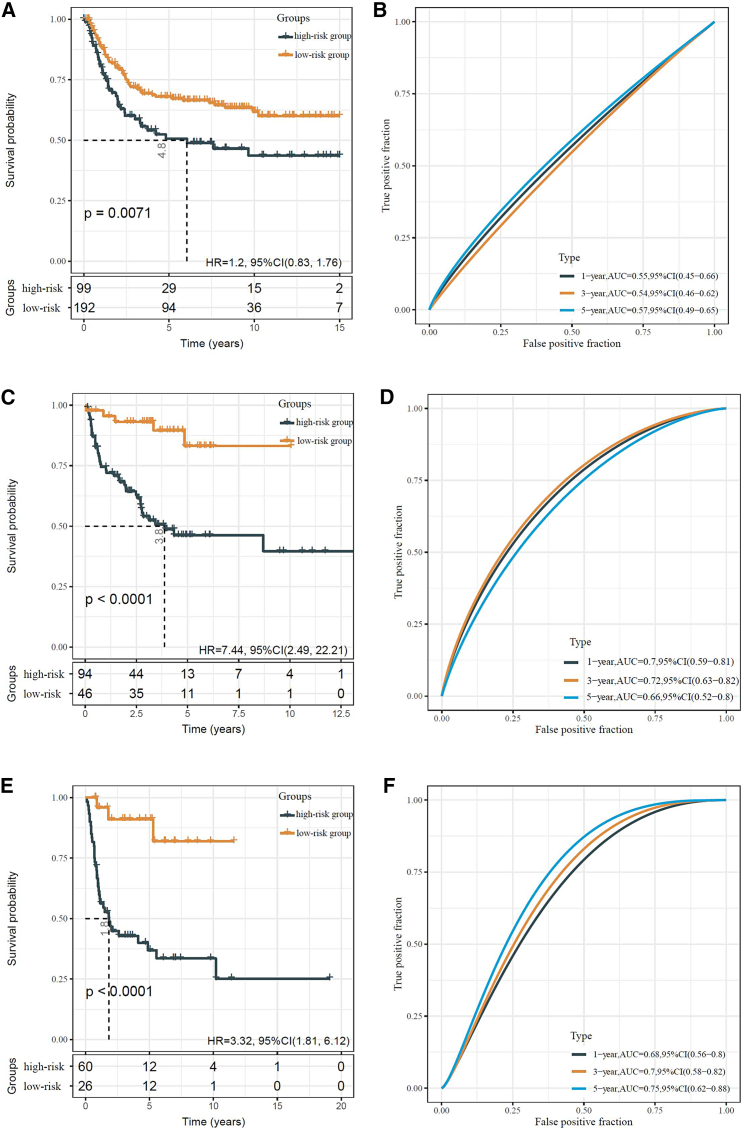
Validation of the DFS-related prognostic relevance of the IRG signature with three external datasets (A) Kaplan–Meier analysis of DFS in the GEO: GSE71118 cohort (validation cohort IV). (B) The AUC of the risk score predicting the 1-, 3-, and 5-year OS in the GEO: GSE71118 cohort. (C) Kaplan–Meier analysis of OS in the GEO: GSE30929 cohort (validation cohort V). (D) The AUC of the risk score predicting the 1-, 3-, and 5-year DFS in the GEO: GSE30929 cohort (E) Kaplan–Meier analysis of DFS in the GEO: GSE40025 cohort (validation cohort VI). (F) The AUC of the risk score predicting the 1-, 3-, and 5-year DFS in the GEO: GSE40025 cohort.

Moreover, the prognostic potential of the IRG signature for DFS was also validated. In the validation cohorts IV–VI, DFS in the high-risk group patients was significantly worse compared with those in the low-risk group (GEO: GSE71118: HR = 1.2, 95% CI: 0.83–1.76, p = 0.0071; GEO: GSE30929: HR = 7.44, 95% CI: 2.49–22.21, p < 0.0001; GEO: GSE40025: HR = 3.32, 95% CI: 1.81–6.12, p < 0.0001; Figures 7A, 7C, and 7E, respectively). Likewise, the predicative accuracy for DFS yielded a valuable 1-, 3-, and 5-year AUC in the three independent validation cohorts (GEO: GSE71118: AUCs of 1-, 3-, and 5-year DFSs were 0.55, 0.54, and 0.57, respectively; GEO: GSE30929: AUCs of 1-, 3-, and 5-year DFS were 0.7, 0.72, and 0.66, respectively; GEO: GSE40025: AUCs of 1-, 3-, and 5-year DFSs were 0.68, 0.7, and 0.75, respectively; Figures 7B, 7D, and 7F, respectively).

### The IRG signature serves as a potential indicator for immune characteristics and immunotherapy response

**Figure 8:**
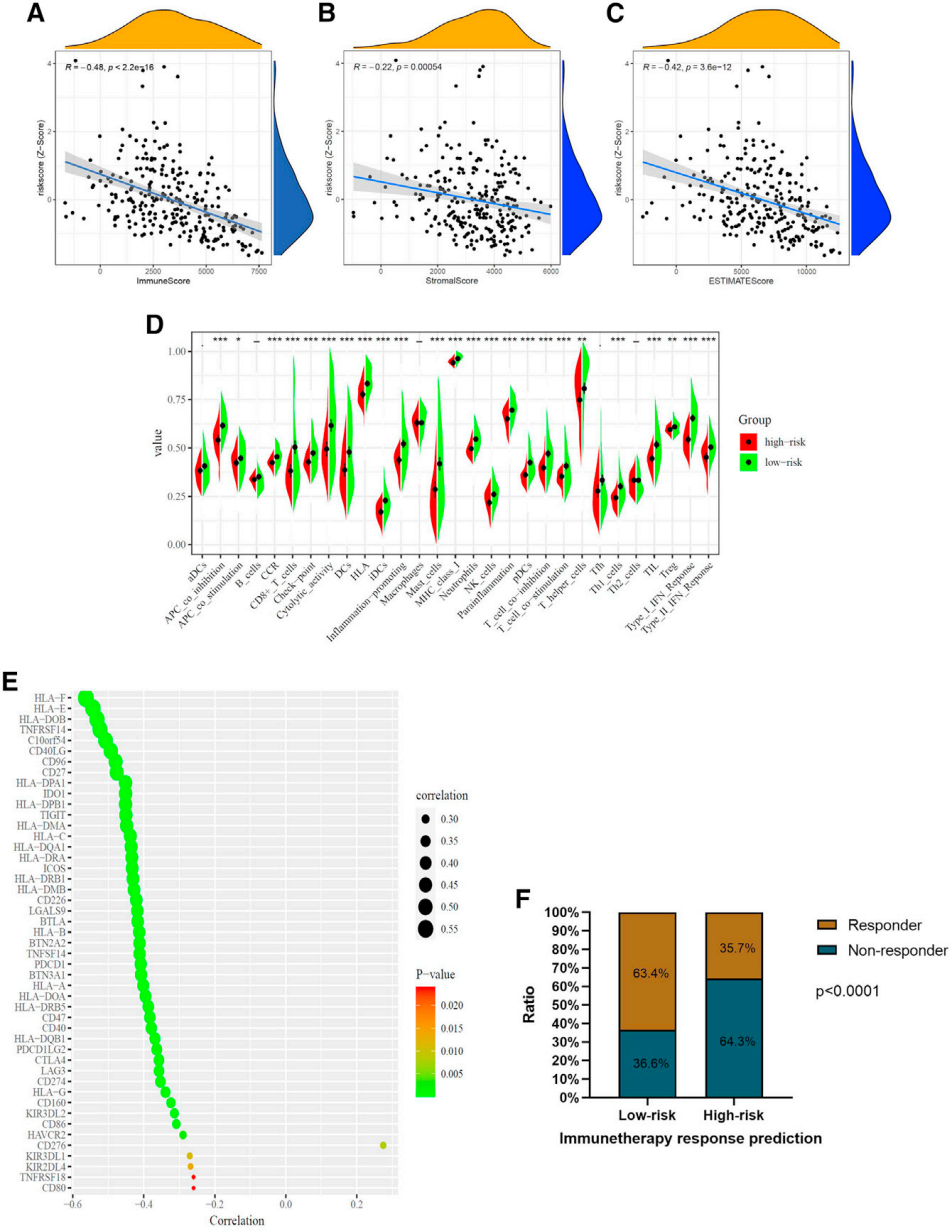
Immune microenvironment analysis and immunotherapy response prediction (A–C) The correlation analysis indicated that the immune score (R = −0.48; p < 0.001) (A), the stromal score (R = −0.22; p < 0.001) (B), and the ESTIMATE score (R = −0.42; p < 0.001) (C) were significantly negatively correlated with the *Z* score of the IRG signature risk score. (D) 29 ssGSEA enrichment levels of the immune signatures in the high- and low-risk groups. (E) The correlation analysis bubble diagram showed that 46 immune checkpoint genes were negatively correlated with the risk score. (F) The ratio of immunotherapy response is significantly increased in the low-risk group compared with the high-risk group. ssGSEA, single sample gene set enrichment analysis.

Since the 14-gene signature was closely related to immune cell abundance scores, the difference of immunological features and immunotherapy responses in high- and low-risk groups were investigated further. For this purpose, the ESTIMATE (estimation of stromal and immune cells in malignant tumor tissues using expression data) algorithm was applied to assess tumor purity and the ratio of infiltrating stromal/immune cells in tumor tissues based on gene expression profiles. The correlation analysis revealed that the risk score of the IRG signature was significantly negatively correlated with the immune score (R = −0.48, p < 0.0001), the stromal score (R = −0.22, p = 0.00054), and the ESTIMATE score (R = −0.42, p < 0.0001) (Figures 8A–8C).

Next, the single sample gene set enrichment analysis (ssGSEA) scores were used to assess the immunogenic activity in the sarcoma samples based on the 29 gene sets representing diverse immune cell types, functions, and pathways. The low-risk group showed a higher immune cell infiltration level compared with the high-risk group (Figure 6D). More importantly, there was a significant negative correlation between the IRG risk score and the expression levels of most immune checkpoint genes (ICGs) (Figure 8E). Furthermore, the immunotherapy response ratio between high- and low-risk groups was calculated by the “ImmuCellAI” online tool (http://bioinfo.life.hust.edu.cn/ImmuCellAI#!/). As shown in Figure 8F, 63.4% of patients in the low-risk group were sensitive to immunotherapy, which is significantly more patients compared with those in the high-risk group (p < 0.0001).

### Nomogram and decision tree based on the IRG signature

**Figure 9:**
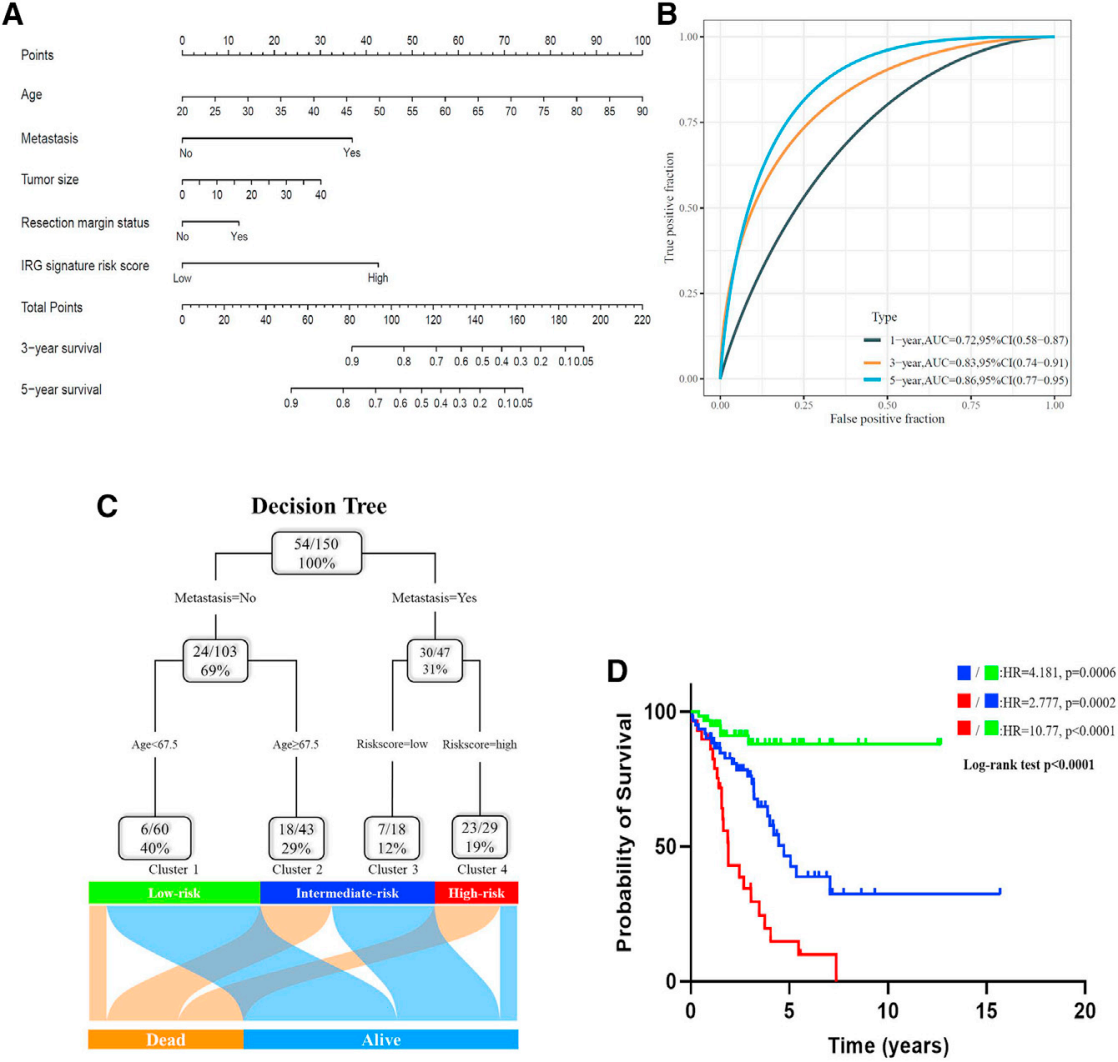
Combination of the IRG signature risk score and clinicopathological features improves prognosis prediction and risk stratification (A) A nomogram was established to predict 3- and 5-year OS in individual sarcoma patients. (B) The AUC of the nomogram to predict the 3- and 5-year OS in the TCGA-SARC cohort. (C) A decision tree was constructed to categorize patients into three different risk levels. (D) Kaplan–Meier analysis of OS of the three different subgroups.

In order to better apply the newly generated IRG signature to clinical practice, a nomogram for predicting OS was developed (Figure 9A). For this analysis, 150 patients of the TCGA-SARC cohort with complete clinicopathological information were used, and the risk score was incorporated additionally. The nomogram included various clinicopathological information (age, metastasis, tumor size, resection margin) and the risk score derived from the IRG signature. The integrated nomogram showed a high predictive accuracy for OS with a calculated 1-year AUC of 0.72 (95% CI: 0.58–0.87), a 3-year AUC of 0.83 (95% CI: 0.74–0.91), and a 5-year AUC of 0.86 (95% CI: 0.77–0.95), respectively (Figure 9B). The calibration plots of observed and predicted probabilities of 3- and 5-year OS presented a good consistency (Figure S3A). Moreover, the decision curve analysis (DCA) showed that the net benefit of the nomogram was superior to a model built with traditional clinicopathological information (Figure S3B).

To further optimize the risk stratification for sarcoma patients, a recursive partitioning analysis was done, and a decision tree for OS was established. Finally, the IRG signature risk score along with age and distant metastasis remained in the decision tree, and three different risk subgroups were identified (Figure 9C). The Kaplan–Meier analysis proved the prognostic relevance of the established decision tree and showed a significant difference in OS among the three subgroups (overall: p < 0.0001; high risk versus intermediate risk: p = 0.0002, HR = 2.777, 95% CI: 1.44–5.34; intermediate risk versus low risk: p = 0.0006, HR = 4.181, 95% CI: 2.07–8.45; high risk versus low risk: p < 0.0001, HR = 10.77, 95% CI: 4.71–24.64; Figure 9D). In order to compare with the risk stratification based on the traditional clinicopathological information alone, the second decision tree was developed, which contains three decision nodes of metastasis, age, and tumor size (Figure S4A). The survival analysis also demonstrated a significant difference in OS among the three risk subgroups generated by the second decision tree (overall: p < 0.0001; high risk versus intermediate risk: p = 0.0002, HR = 3.346, 95% CI: 1.19–9.40; intermediate risk versus low risk: p < 0.0001, HR = 5.191, 95% CI: 2.83–9.52; high risk versus low risk: p < 0.0001, HR = 12.85, 95% CI: 3.05–54.14; Figure S4B).

## Discussion

Sarcomas are a heterogeneous malignant disease of mesenchymal origin, which account for approximately 1% of malignant tumors in adults and 15% of all children malignancies.^18^ The survival rates of each sarcoma subtype differ due to disease heterogeneity. However, establishing appropriate prognostic biomarkers is critical for precision medicine and risk stratification. In this respect, recent studies demonstrated that distinct immune gene signatures are prognostically relevant in cancer patients.^19^, ^20^, ^21^, ^22^ However, the IRGs selected in most studies are derived from the ImmuPort database or from ssGSEA. In the present study, the 14-IRG signature for sarcoma patients is based on ImmucellAI and WGCNA, which provides a novel and innovative method to identify prognostic biomarkers. Moreover, the newly presented IRG signature proved to act as a powerful and accurate prognostic parameter in seven different sarcoma cohorts. Furthermore, the risk score derived from the IRG signature was significantly correlated with the immune microenvironment and the response to immune checkpoint therapy. In order to quantify the risk assessment of each patient, a nomogram and a decision tree integrating the IRG signature risk score were established. Both proved to serve as valuable and robust predictive tools for the survival outcomes of sarcoma patients.

In the present study, a significant prognostic correlation between a high IRG signature score and shorter OS and DFS in seven different sarcoma cohorts was demonstrated. This is the most comprehensive study including the biggest collective of sarcoma patients analyzing seven public sarcoma datasets with almost 1,000 patients. In the developed 14-IRG signature, the expression level of twelve genes (TRIM21, TNF, CRIP1, FCER1A, SLC25A20, ZNFX1, DHX58, TNFSF15, TAPBPL, CMA1, APOL2, and GBP2) were negatively correlated with OS. TRIM21 (tripartite motif-containing protein 21) seems to be the most powerful gene since it had the most negative coefficient value. TRIM21 is involved in innate immunity and cancer progression. Previous studies found that the overexpression of TRIM21 inhibits tumor migration and invasion in breast cancer cell lines. Correspondingly, high TRIM21 expression rates were associated with increased survival rates of breast cancer patients.^23^^,^^24^ However, Zeng et al. reported that TRIM21 positively regulated osteosarcoma cell proliferation and autophagy.^25^ Only two genes (NT5DC2 and REC8) act as oncogenes in the IRG signature. NT5DC2 (5′-nucleotidase domain-containing 2) is the most significant risk gene with a high coefficient. It has been shown to be involved in tumor development and progression in various types of carcinomas, such as colorectal cancer, lung cancer, hepatocellular carcinoma, and glioma.^26^, ^27^, ^28^, ^29^ Nonetheless, only little is known about the exact function of NT5DC2 in sarcomas. In this respect, the present study firstly reported the potential prognostic value of NT5DC2 in sarcoma patients. Additionally, recent findings further support the present study. Hu et al. demonstrated that the overexpression of NT5DC2 promotes leiomyosarcoma progression and proliferation both *in vitro* and *in vivo*.^30^ Therefore, NT5DC2 may be a novel biomarker and therapeutic target for sarcomas, but further studies are needed to verify its biological function and prognostic value.

Notably, the risk score of the IRG signature demonstrated a significant predictive capacity for survival outcomes in the training set and in all validation cohorts. A multivariate regression analysis showed that the IRG signature risk score was an independent risk factor for both OS and DFS. Moreover, the AUC of the IRG risk score for 3-year OS was 0.8, which is outperforming previously published genetic risk scores.^31^ These findings indicate that the 14-gene signature may play a critical role in the development and progression of sarcomas and may serve as a prognostic tool. In contrast to previous studies,^32^, ^33^, ^34^ the novel IRG signature proved to be suitable for different sarcoma subtypes. The present results suggest that although sarcoma subtypes are partially heterogeneous, these entities share a common genetic background. Therefore, it is necessary to design further experimental research to investigate the biological function of the identified 14 genes in different sarcoma subtypes.

In this respect, adjuvant^35^, ^36^, ^37^ and neoadjuvant^38^, ^39^, ^40^ systemic therapies reduce the risk of metastatic spread significantly, subsequently leading to prolonged OS. Nonetheless, the findings of the individual trials are sometimes inconclusive, and treatment regimens differ. Recently, it has been demonstrated that a risk-adjusted chemotherapy is associated with significantly increased survival rates.^41^ The risk adjustment was done with a nomogram, which was based only on clinicopathological parameters like age, tumor size, and histology, particularly disregarding the tumor biology.^41^^,^^42^ However, a histotype-tailored neoadjuvant chemotherapy had no prognostic benefit in a multicenter trial.^38^ Thus, the identification of patients at risk is of utmost importance to design further studies investigating multimodal therapy. The present findings suggest that the introduced nomogram and the IRG signature might be appropriate tools to stratify patients.

In an attempt to better apply the IRG signature in a clinical setting, a nomogram and a novel decision tree were established. The results of the AUC, calibration curve, and decision curve analysis (DCA) demonstrated that the nomogram including the IRG signature risk score is a powerful and accurate predictive tool for sarcoma patients. Two previously published studies also created similar predictive nomograms based on clinicopathological features or immunoscoring. However, these studies evaluated the prognostic value in only two different sarcoma cohorts and reached lower AUC values compared with the present manuscript.^43^^,^^44^ Aside from the new IRG signature risk score, the nomogram contains established prognostic factors (age, metastasis, tumor size, and resection margin status).^45^ However, due to missing data, the present nomogram did not include tumor grade, which has been identified as the most significant independent prognostic factor so far. Nonetheless, adding the IRG signature to the nomogram improved its predictive value significantly. To further improve the performance of predictive models, future studies need to add the IRG signature to their nomograms.

The decision tree was established to optimize risk stratification for OS in sarcoma patients. Finally, the decision tree was built including three components: metastasis acted as the most important determinant; age (cut-off point: 67.5 years) and the IRG risk score were additional factors. During the trimming step of the decision tree, it showed a strong relationship between the IRG signature risk score and high-risk sarcoma patients. The grouping results of the decision tree showed significant differences in the survival curves among the three different risk subgroups, demonstrating its great performance in patient stratification. Thus, using a simple clinical decision tree enables clinicians to identify patients with poor prognoses efficiently and accurately. Interestingly, the risk stratification generated by the IRG-risk-score-based decision tree model has no significant benefit compared with the model based on clinicopathological characteristics alone. However, the decision tree based on the IRG risk score represents a better predictive model because it is not only easier to interpret the threshold of the decision nodes from a medical point of view but also the structure is simpler to avoid model overfitting.

In general, sarcomas are not highly immunogenic tumors. Nonetheless, in previous studies, immune checkpoint inhibitors (ICIs) exhibited antitumor effects in 5%–40% of sarcoma patients.^46^, ^47^, ^48^, ^49^, ^50^ In the present study, the IRG risk score was negatively correlated with the immune score and the expression levels of ICGs. A previously published study revealed that the expression of PD-L1 was associated with more PD-1-positive TILs in soft-tissue sarcomas (STSs).^51^ Additionally, the increased TIL counts due to neoadjuvant therapy were beneficial and seemed to prolong DFS of STS patients.^52^ The findings of the present study indicate that low-risk sarcomas with an increased immune cell infiltration have a higher immunogenicity and longer survival time. Furthermore, the prediction model for a response to immunotherapy indicates that low-risk sarcomas are more sensitive to immunotherapy. Taken together, these results underpin that the newly introduced IRG signature may serve as a biomarker to predict responses to ICIs. Nonetheless, this has to be shown in prospective trials. Thus, the IRG signature provides an additional opportunity to identify patients with sarcomas eligible for ICI therapy, which has to be addressed in future studies.

Although this study established a powerful IRG signature, a corresponding nomogram, and a decision tree for sarcoma patients, there are limitations as well. Firstly, owing to the rarity of sarcomas, a profound investigation of each sarcoma subtype is difficult. Thus, the present study includes all available subtypes of sarcomas introducing heterogeneity. Nonetheless, the IRG risk signature proved to exert a prognostic benefit in every subtype. Thus, further multicenter studies are highly demanded to analyze the risk signature in larger cohorts in a clinical setting. Secondly, the established nomogram and the decision tree were based on one training cohort (TCGA-SARC) and have not been tested in other validation cohorts due to incomplete clinical data. Furthermore, the ratio of immunotherapy responses was estimated *in silico*, which requires more reliable cohort studies in real-world. Nonetheless, this theoretical approach has been successfully validated in several solid tumor cohorts, such as melanoma, lung cancer, bladder cancer, and gastric cancer.^17^

In conclusion, using a WGCNA and a Lasso cox regression based on the abundance score of immune cells in sarcomas, a novel 14-IRG signature has been identified to be prognostically relevant. This signature serves as a robust and independent prognostic biomarker in various sarcoma subtypes. In addition, the present study developed a nomogram and a decision tree based on this IRG signature, which potentially act as an accurate and practical predictive tool to identify high-risk patients with low survival rates. Moreover, the IRG signature also had a reliable ability to predict the response to immunotherapy and may help to improve the efficacy of personalized immunotherapy in sarcoma patients.

## Materials and methods

### Data acquiring and preprocessing

Sarcoma-related clinical data and RNA sequencing (RNA-seq) data from TCGA-SARC were obtained from the UCSC Xena browser (https://xenabrowser.net/) and were used in the training set. Patients with missing information about survival time, survival status, and clinicopathological characteristics were excluded. In total, 256 sarcoma patients with complete survival data and gene expression profiles were included in the training set. The information about age, sex, resection margin status, tumor size, and radiation therapy was complete for 150 patients. These 150 patients were used for establishing the nomogram and the decision tree analysis.

Six validation groups were established, and the data was downloaded from two databases. The clinical data and gene expression files of 85 osteosarcoma patients derived from TARGET-Osteosarcoma (https://ocg.cancer.gov/programs/target/projects/osteosarcoma) were used as the first validation cohort. Moreover, the clinical information and gene expression data of five additional independent validation cohorts (GEO: GSE17674: 45 Ewing sarcoma patients; GEO: GSE119041: 50 uterine sarcoma patients; GEO: GSE71118: 291 various sarcoma patients; GEO: GSE30929: 140 liposarcoma patients; and GEO: GSE40025: 86 synovial sarcoma patients) were downloaded from GEO (http://www.ncbi.nlm.nih.gov/geo/). Among them, the TARGET-Osteosarcoma GEO: GSE17674 and GSE119041 cohorts were used to verify the OS, while GEO: GSE71118, GSE30929, and GSE40025 cohorts were used to validate the DFS. All RNA-seq and microarray data included in the present study were normalized and log_2_(X+1)-transformed.

### Gene selection and signature establishment

The infiltration score of each sample and the abundance score of 24 immune cell types including 18 T cell subtypes (CD4+, CD8+, CD4+ naive, CD8+ naive, Tcm, Tem, Tr1, induced regulatory T [iTreg], natural regulatory T [nTreg], Th1, Th2, Th17, Tfh, Tc, mucosal-associated invariant T [MAIT], exhausted T (Tex), gamma delta T (γδ T), and NKT cells) and six other immune cells (B cell, NK cells, monocytes, macrophages, neutrophils, dendritic cells [DCs]) of TCGA-SARC were downloaded from the ImmuCellAI database. A univariate Cox proportional hazard regression analysis was performed to determine the prognostic value of the infiltration score of the different immune cells in the TCGA-SARC cohort using the R package “survival.” Then, the gene co-expression network was constructed by the R package “WGCNA” using whole-transcriptome profiling data.^53^ The threshold for the determination of weighted adjacency matrix was fixed at a soft power of 5 and a scale-free R2 >0.85, respectively.

In a next step, the topological matrix was constructed by using the topological overlap measure (TOM) in the R project. The minimum module size was 80 genes. The featured genes of the modules were calculated, and the similar modules were clustered and merged according to the module dissection threshold. Finally, module trait co-expression similarity and adjacency analyses were performed in the identified gene modules. Thereby, the module with the strongest correlation to the abundance score of the prognosis-related immune cells was identified for further analysis. With a threshold of the p value of univariate Cox regression <0.05, 220 candidate genes with prognostic values were extracted from the “MEred module.” Next, the LASSO Cox regression analysis was applied to select the most powerful prognostic genes of the “MEred module” with R package “glmnet.” The penalty regularization parameter λ was fixed using a 10-fold cross-validation to prevent an overfitting effect. The risk score of the IRG signature was calculated as follows:

$$Risk\;score=\underset{i}{\sum }Coefficient\left(mRNA_{i}\right)\times Expression\left(mRNA_{i}\right)\text{,}$$

where *i* is the selected gene in the IRG signature. Patients were classified into high- and low-risk groups according to the optimal cut-off value, which was determined by the surv_cutpoint function of the R package “survminer.”

### Analysis of immune function and response to immunotherapy

The scores of infiltrating stromal, immune cells, and tumor purity were calculated using the “ESTIMATE” package in R.^54^ The ssGSEA algorithm was applied to comprehensively evaluate the immunological features and the infiltration of immune cells in the TCGA-SARC cohort.^55^ The R packages (“GSVA,” “limma,” and “GSEABase”) were employed to analyze the gene expression profiles of sarcoma samples. Previously, 79 ICGs have been demonstrated to reliably be related to the response to immunotherapy. In order to analyze the response to immunotherapy, these 79 ICGs have been used in the present analysis.^56^ The immunotherapy response prediction was based on the transcriptional data via the accurate and reliable online tool “ImmuCellAI.”^17^

### Construction of the nomogram and the decision tree

A nomogram integrating clinical information and the immune-related risk signature was established using the R package “rms” to predict the survival probability for each patient.^57^ Calibration curves of observed and predicted probabilities of 3- and 5-year OS were plotted to determine the discrimination of the nomogram. The decision tree was developed for risk stratification via a recursive partitioning analysis (RPA) in the R package “rpart.”^58^

### Bioinformatic and statistical analyses

R software v.4.0.0 (R Foundation for Statistical Computing, Vienna, Austria) and GraphPad Prism 8.4 (GraphPad Software, San Diego, CA, USA) were used to analyze data and plot figures. Univariate and multivariate Cox regression analyses were performed by the corresponding R packages. The Kaplan–Meier method and log rank tests were used to evaluate the difference of survival outcomes between different risk groups with the R package “survminer” and “survival.” The AUC derived from a time-dependent ROC (tROC) analysis was used to quantify the predictive power with the R package “survivalROC.”^59^ The HR and 95% confidence interval (CI) were calculated using log rank tests to confirm the risk score associated with survival time. Student’s t tests or one-way ANOVAs were used for the comparison of continuous variables between groups. A p value < 0.05 (two-tailed) was considered to indicate statistical significance, and high significance was indicated by a p value < 0.001.

## Supplemental information

Document S1. Figures S1–S4

Document S2. Article plus supplemental information
