# Supplementary material for: A novel immune-related gene signature predicting survival in sarcoma patients
Source: Mol Ther Oncolytics. 2021 Dec 9;24:114–26. doi: 10.1016/j.omto.2021.12.007 (PMC8718575; doi:10.1016/j.omto.2021.12.007)
Supplement: Document S1. Figures S1–S4 [file mmc1.pdf]

OMTO, Volume *24*

Supplemental information

A novel immune-related gene signature predicting survival in sarcoma patients

Haoyu Ren, Alexandr V. Bazhin, Elise Pretzsch, Sven Jacob, Haochen Yu, Jiang Zhu, Markus Albertsmeier, Lars H. Lindner, Thomas Knösel, Jens Werner, Martin K. Angele, and Florian Bösch

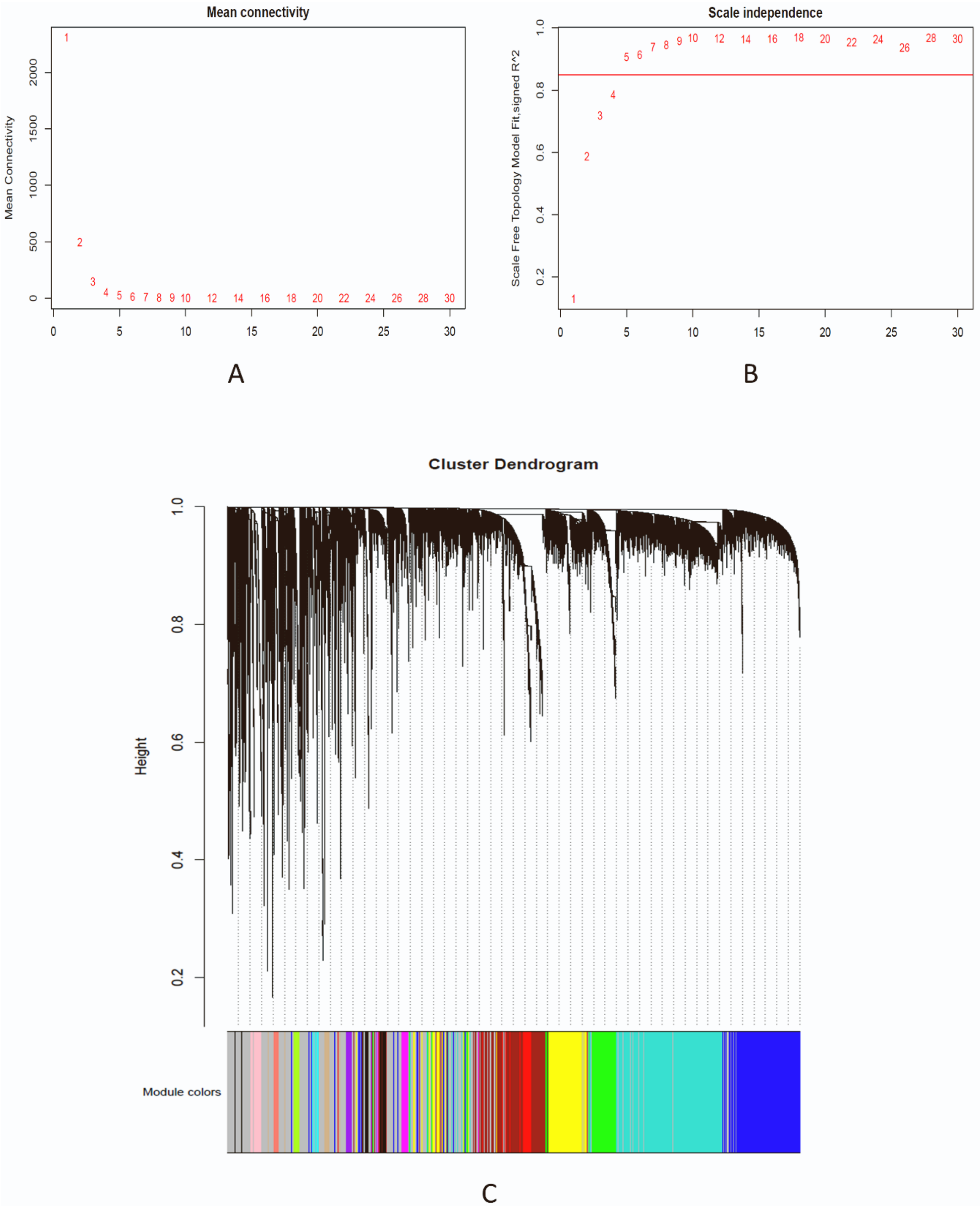

Figure S1. The establishment of a gene co-expression network. (A, B) Soft-thresholding power analysis was used to obtain the scale-free fit index of the network topology. (C) Identification of co-expression modules in TCGA-SARC.

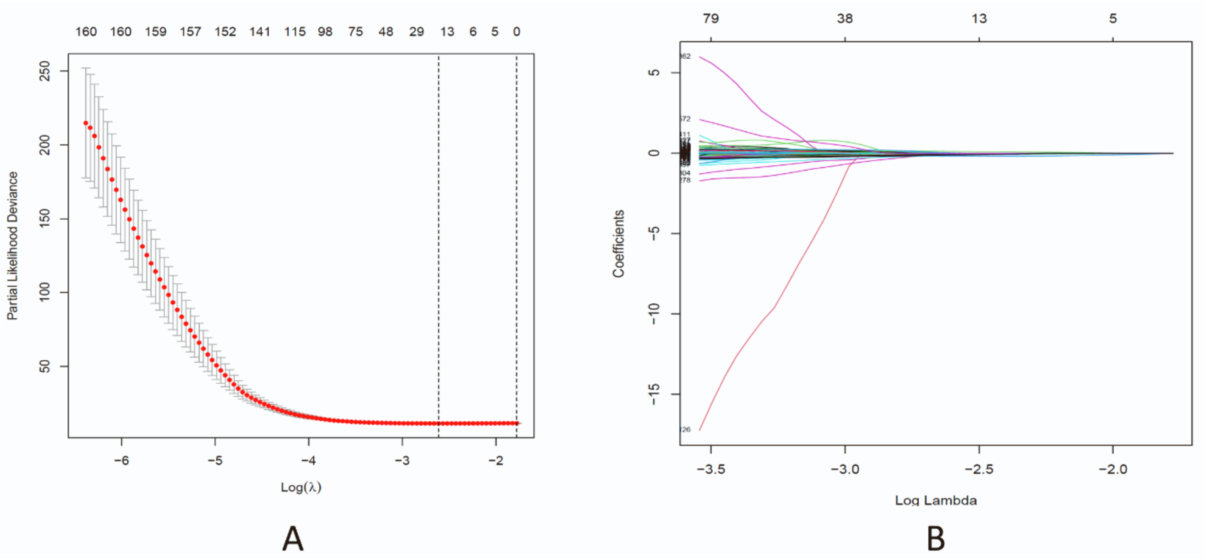

Figure S2. LASSO COX regression analysis for prognostic IRGs. (A) Lambda selection by the partial likelihood deviance. (B) The penalty coefficient of 220 candidate genes was optimized through 10-fold cross-validation in the training set.

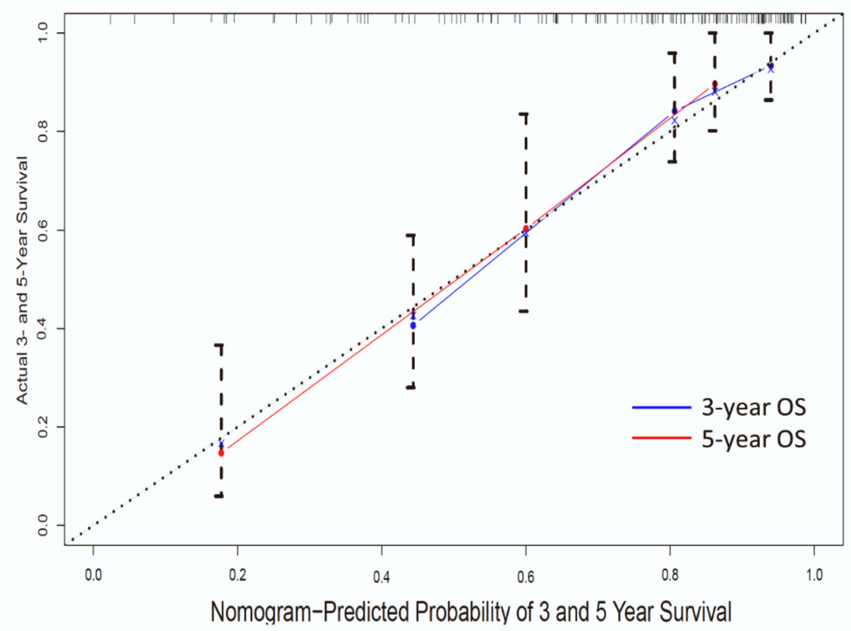

A

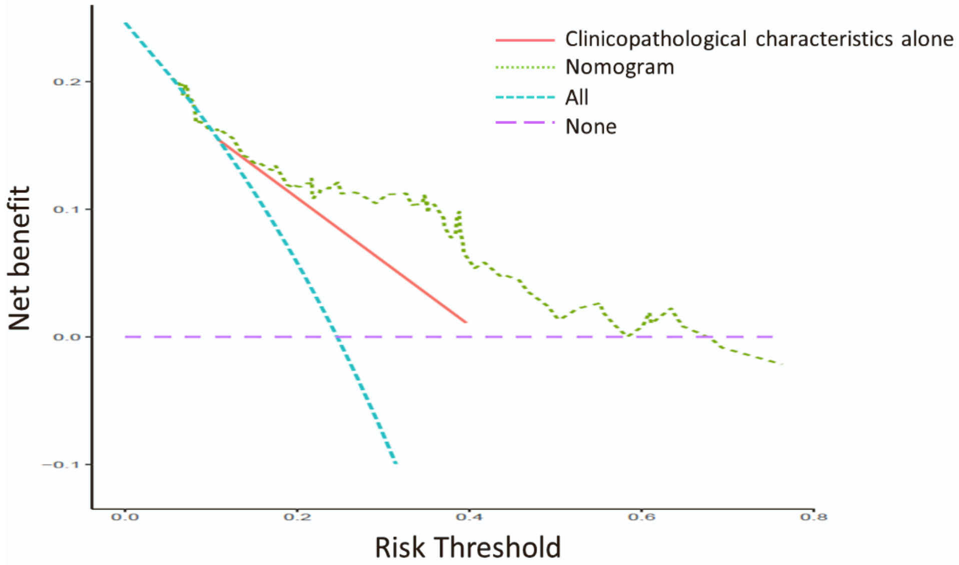

B

Figure S3. The evaluation of the IRS signature risk score-based nomogram. (A) Calibration analysis indicated a high accuracy of 3- and 5-year survival prediction. (B) Decision curve analysis of nomogram and clinicopathological features alone in terms of overall survival in TCGA-SARC. The x-axis represents the threshold probability, and the y-axis shows the net benefit.

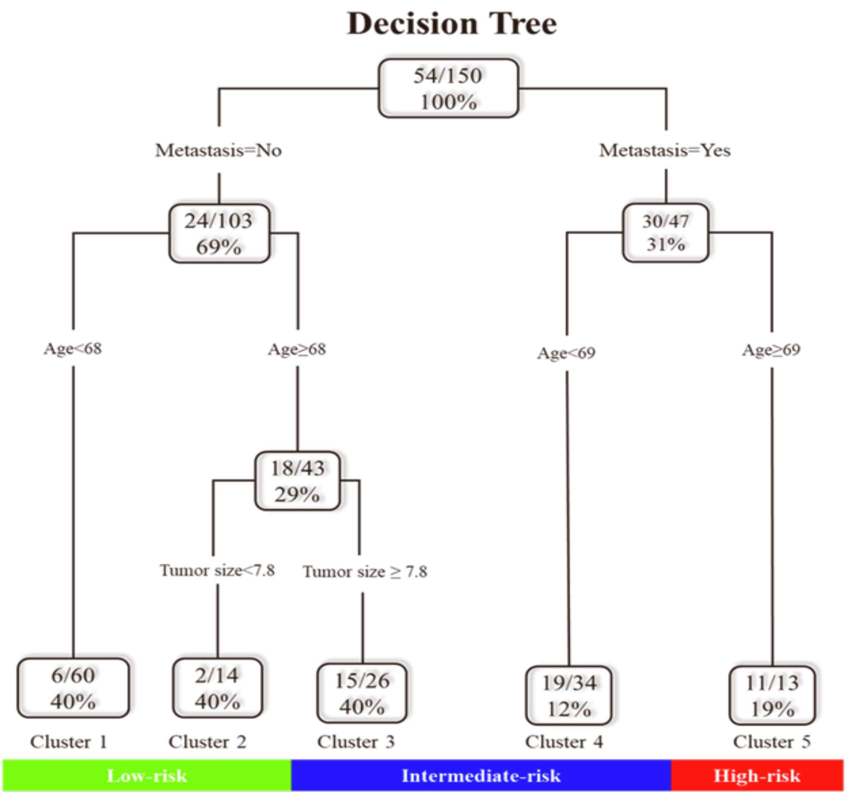

A

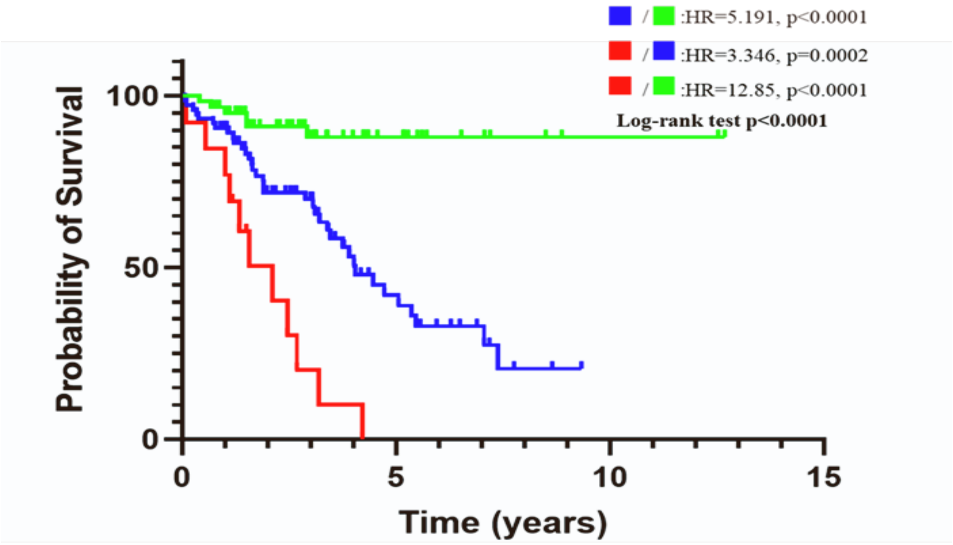

B

Figure S4. The risk stratification based on clinicopathological information alone. (A) A decision tree based on clinicopathological information alone was established to categorize patients into three different risk levels. (B) Kaplan-Meier analysis of overall survival of the three different subgroups.
